# Editorial: Cell and Developmental Signalling in Neuroblastoma

**DOI:** 10.3389/fcell.2022.1126352

**Published:** 2023-01-04

**Authors:** Rafael Pulido, Caroline E. Nunes-Xavier

**Affiliations:** ^1^ Biomarkers in Cancer, Biocruces Bizkaia Health Research Institute, Barakaldo, Spain; ^2^ Ikerbasque, Basque Foundation for Science, Bilbao, Spain; ^3^ Department of Tumor Biology, Institute for Cancer Research, The Norwegian Radium Hospital, Oslo University Hospital, Oslo, Norway

**Keywords:** neuroblastoma, cell signaling, cell differentiation, kinase, phosphatase, super-enhancer, retinoic acid

Neuroblastoma is a heterogeneous embryonal malignancy that usually manifests in the infancy because of alterations in the normal development of cells derived from the neural crest, which affect the speciation of the cell populations from the sympathetic nervous system, including the adrenal gland and paraspinal sympathetic ganglia. Neuroblastoma can be considered as a developmental disease where the tight regulation of growth and differentiation of precursor neuroendocrine cells, as well as the surveillance and activity of cancer stem cells, play major roles in its initiation and maintenance. In summary, cell signal transduction in response to spatial and temporal external cues orchestrates the regulation of gene expression pattern networks during neuroendocrine cell development, and alterations in these regulatory pathways lead to neuroblastoma manifestation. Although neuroblastoma share with other pediatric cancers a low mutational burden, some specific genes are targeted for somatic mutation with relative high frequency, among which the tyrosine kinase receptor *ALK* and the tyrosine phosphatase *PTPN11* genes are illustrative examples. In addition, germline mutations in *ALK* or in the neuronal differentiation transcription factor *PHOX2B* gene are causative of familial neuroblastoma. Chromosomal alterations are frequent in neuroblastoma, with *MYCN* amplification, 17q gain, and 11q and 1p loss, among others, being detected in many patients, usually in association with high-risk prognosis. Chromosomal rearrangements affecting the promoter of the telomerase *TERT* gene, resulting in chromatin remodeling, are also frequent. Many recent reviews provide excellent accounts of neuroblastoma origin, biology, and development. Because of brevity, only some of them are referenced here (Aygun, 2018; Tomolonis et al., 2018; Tsubota and Kadomatsu, 2018; Takita, 2021; Lundberg et al., 2022; Ponzoni et al., 2022).

Neuroblastoma is a rare cancer, with an overall incidence of 2–4 cases per year per million inhabitants, although neuroblastoma incidence in children population reaches an overall estimation of 1 case per year per 10,000 children, being one of the most common cancer in infants. Survival rates of neuroblastoma patients have increased in the last years, oscillating between 95% 5-year survival rate for low-risk group to 50% 5-year survival rate for high-risk group. The major criteria to include patients in the high-risk group is age at diagnosis, *MYCN* amplification, and chromosomal alterations (https://www.cancer.org/cancer/neuroblastoma/). Current standards for treatment of these patients include combinatorial chemotherapy, surgery, autologous stem cell rescue, radiation therapy, 13-cis-retinoic acid maintenance therapy, and anti-GD2 targeted disialoganglioside therapy. Clinical assays targeting signalling proteins, such as ALK, VEGF, or mTOR, among others, are ongoing (Zafar et al., 2021). Dedicated research to improve the efficacy of therapies for patients with high-risk neuroblastoma patients is a necessity, including molecular and cellular studies to understand therapy resistance and recurrence.

This Research Topic has addressed the analysis of cell and developmental signalling processes which may impact on the initiation and progression of human neuroblastoma. An overview of cell signalling in neuroblastoma is provided by Nunes-Xavier et al. with a focus on protein tyrosine phosphatases (PTP) as novel effectors and potential biomarkers and therapeutic targets in neuroblastoma. These PTP include PTPN11, a major regulator of the RAS/MAPK pathway whose expression associates with bad prognosis and whose pharmacological inhibition has been proposed as an alternative anti-neuroblastoma therapy. In addition, other dual-specificity PTP which directly regulate MAPK have been associated with the outcome of neuroblastoma patients (Nunes-Xavier et al., 2019). As mentioned, chromosomal imbalances are major indicators of neuroblastoma prognosis, and the importance and mechanisms of chromosome instability in neuroblastoma has been reviewed by Paolini et al., with the proposal of a multi-step model of neuroblastoma progression based on the progressive development of chromosome segmental alterations. The identification of the specific genes and non-coding RNAs responsible of the neuroblastoma pathogenicity associated with loss and gain of chromosomal regions constitute a priority in the field. Inappropriate differentiation of neuroendocrine cell precursors is an evinced model for neuroblastoma development. Parkinson et al. report the dual role of the proneural master regulator of transcription, ASCL1, in maintaining proliferation and priming differentiation of neuroblastoma cells. Further insights into neuroblastoma cell differentiation by retinoic acid, a pro-differentiating neuroblastoma agent in neuroblastoma maintenance therapy (Bayeva et al., 2021), are provided by Gomez et al. These authors analysed the expression in neuroblastoma cells of transcription factors from core regulatory circuitries under super-enhancers regulation, in response to retinoic acid.

Immunotherapy based on direct immune checkpoint inhibition is providing a consistent improvement in the treatment of several solid cancers, but this is not the case of high-risk neuroblastoma. Alternative passive and active immunotherapy approaches, including novel CAR-T/NK therapies, are being tested in high-risk neuroblastoma patients, with a focus on molecules highly expressed on the surface of neuroblastoma cells, such as the GD2 disialoganglioside and B7-H3 immune checkpoint protein (Nguyen and Thiele, 2021; Anderson et al., 2022; Pulido and Nunes-Xavier, 2023). Understanding cell and developmental signaling that specifically operates in neuroblastoma will help in the optimization of novel and more effective anti-neuroblastoma therapies.
